# Synthesis and characterization of novel donor–acceptor type neutral green electrochromic polymers containing an indolo[3,2-*b*]carbazole donor and diketopyrrolopyrrole acceptor†

**DOI:** 10.1039/c8ra03552k

**Published:** 2018-06-11

**Authors:** Yan Zhang, Lingqian Kong, Xiuping Ju, Hongmei Du, Jinsheng Zhao, Yu Xie

**Affiliations:** Shandong Key Laboratory of Chemical Energy Storage and Novel Cell Technology, Liaocheng University Liaocheng 252059 P. R. China j.s.zhao@163.com; Dongchang College, Liaocheng University Liaocheng 252059 P. R. China; College of Environment and Chemical Engineering, Nanchang Hangkong University Nanchang 330063 PR China xieyu_121@163.com

## Abstract

Indolocarbazole bearing donor–acceptor type polymers have rarely been reported in the electrochromic field despite them having considerable development in the applications of organic photoelectric devices. In this paper, two novel soluble electrochromic polymers, namely PDTCZ-1 and PDTCZ-2, were prepared by chemical polymerization including indolo[3,2-*b*]carbazole (IC) units as the donor, diketopyrrolopyrrole (DPP) units as the acceptor and bithiophene units as the bridging group. Through diverse characterization techniques such as cyclic voltammetry (CV), scanning electron microscopy (SEM), UV-vis spectroscopy and thermogravimetric analysis (TGA), it was found that PDTCZ-1 and PDTCZ-2 exhibited saturated green in the neutral state and pale green in the oxidized state with optical band gaps of 1.44 eV and 1.39 eV, respectively, as well as demonstrating fast switching speed, satisfactory coloration efficiency and favorable thermal stability. In addition, the proportion of donors to acceptors definitely exerted an influence on the electrochromic properties of the polymers. As the thiophene/IC/DPP ratio changed from 4/3/1 (PDTCZ-1) to 5/4/1 (PDTCZ-2), meaning an increase of the donor ratio, the polymer showed a reduced onset oxidation potential, decreased optical band gap and different dynamic parameters. The positive results suggest that PDTCZ-1 and PDTCZ-2 could be promising candidates as neutral green electrochromic materials and deserve more attention and penetrating research.

## Abstract 2

Two novel neutral green D–A type conjugated polymers were synthesized, illustrating satisfactory electrochromic properties, such as low band gaps, desirable color switches, excellent solubility and favorable thermal stability.

## Graphical abstract

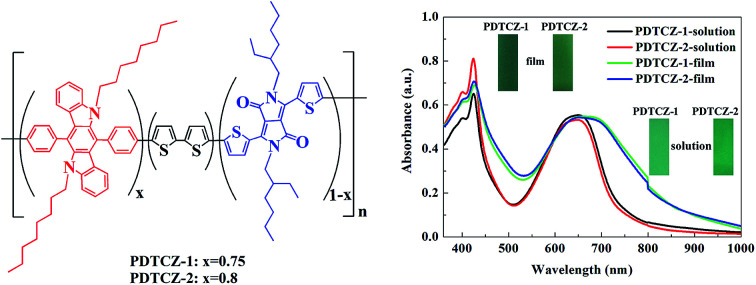

## 1. Introduction

Conducting polymers (CPs), as a class of organic materials with electrical conductivity like semiconductors and metals, possess many attractive advantages including conductivity, favorable mechanical flexibility, good processability, and light weight.^1,2^ In particular, CPs can be nanostructured in order to meet the requirements for some specific applications.^3–5^ Through the unremitting efforts of researchers, both bulk CPs and nanostructured CPs have been widely explored and developed in the fields of chemical sensors, electromagnetic shielding materials, supercapacitors, electrochromic devices, and so on.^6–9^ Electrochromism is considered as a process by which the material undergoes stable and reversible optical change under the applied potentials.^9^ Electrochromic polymers, as an important branch of CPs, have become a research hotspot in recent years due to their characteristics of multiple color changes, short response time, excellent cyclic reversibility, easy molecular structure design, and low cost.^10–12^

Color change is a pivotal feature for electrochromic materials and considered as a crucial objective for research specialist staff.^13,14^ According to the color theory, all types of color can be realized by a combination of three-primary colors namely red, green and blue.^15^ Compared with red and blue, neutral green electrochromic polymers are difficult to achieve since they require two absorption bands located in both red and blue regions at the same time. Besides, for the sake of transparent color in the oxidized state, the two bands should be controlled by the same potentials and disappear simultaneously.^16–19^ Up to now, there are several strategies to adjust the polymer color such as growing conjugate chain, tuning steric interaction and adopting donor–acceptor (D–A) approach, among which synthesizing D–A type polymers is by far the most popular and effective method through designing different donors and acceptors.^16,20^ Furthermore, another significant advantage of D–A structure is the lower band gap of polymer owing to the inter- or intra-molecular charge transfer effects from push/pull interactions between donor and acceptor.^21,22^ Wudl group synthesized the first case of soluble neutral green electrochromic polymer containing thieno[3,4-*b*]pyrazine and thiophene units which showed the band gap as low as 1.3 eV.^23^ Subsequently, Toppare, Reynolds and other research groups investigated various D–A type neutral green electrochromic polymers mainly based on benzothiadiazole^24,25^ and quinoxaline^19,26^ units with low band gaps. In particular, the investigation on a polymer of poly((2-dodecyl-4,7-di(thiophen-2-yl)-2*H*-benzo[*d*][1,2,3]triazole)) (PTBT) can be regarded as an important breakthrough in the multi-colored electrochromic materials over recent years because it showed different colors covering red, green, blue, black and transmissive states with low band gap of 1.65 eV.^27^ Xu group reported two D–A type soluble polymers bearing the modified bay-annulated indigo (BAI) as acceptor for efficient electrochromic devices which changed between deep blue and light green with low band gaps (about 1.5 eV) and superior stability.^28^ Our group also synthesized some neutral green polymers containing [1,2,5]thiadiazolo[3,4-*c*]pyridine,^15^ quinoxaline^29^ and pyrido[3,4-*b*]pyrazine^30^ as acceptor units. Although great efforts have been devoted in research of green electrochromic polymers, the number of this species is still rare and there is expansive space for development in terms of their comprehensive properties and applications.

Various neutral green D–A type polymers previously reported primarily employ thiophene derivatives, especially 3,4-ethoxylenedioxythiophene (EDOT) and 3,4-propylenedioxythiophene (ProDOT) as the donors, which causes the diversity of polymer species depends on changes in acceptors.^16–19,25,29^ So actively exploring new kind of donors can be considered as an effectual way to expand green electrochromic polymers. Indolo[3,2-*b*]carbazole (IC), as an important class of donor unit containing electron-rich indole and carbazole groups, has notable planar and rigid conjugate structures similar with pentacene, which could facilitate charge carrier effectively and result in high hole transporting ability for its corresponding polymers.^31,32^ On the other hand, IC based polymers present excellent photo-stability and environmental stability due to their low-lying highest occupied molecular orbital (HOMO) level.^33,34^ Finally, the structure of IC can be modified at 2,8-positions, 3,9-positions, and 5,11-positions by different functional groups to enhance solubility and tune property of the polymers.^34,35^ The above advantages make IC and its derivatives receive extensive studies in the applications of photovoltaic cells,^31,32^ organic field-effect transistors (OFET)^33–35^ and organic light emitting diodes (OLED).^36,37^ Qian *et al.* prepared several kinds of IC-containing multi-donor–π–acceptor type organic dyes used in dye-sensitized solar cells, among which a polymer named QX02 showed the power conversion efficiency as high as 8.09% which was very close to that of the commercial cell.^32^ Boudreault *et al.* synthesized 3,9-diphenyl-5,11-dioctylindolo[3,2-*b*]carbazole used as the active layer in an OFET with excellent stability and high hole mobility of 0.2 cm^2^ V^−1^ s^−1^.^35^ However, the electrochromic polymers employing IC units as donor have been investigated rarely owing to their poor solubility.^38,39^ Gokce *et al.* prepared a D–A type electrochromic polymer containing IC units namely poly(3,9-(3,3-didecyl-3,4-propylenedioxythiophene)-5,11-dihydroindolo[3,2-*b*]carbazole) which exhibited color change from yellow to cyan in the oxidation process with 1.56 eV band gap, as well as showed capacitance property and favorable solubility in common organic solvents.^38^ Zhang *et al.* studied on an indolo[3,2-*b*]carbazole-based compound with two diphenylamine termini and found it displayed reversible color change from light-yellow to red, and to blue at different oxidation states.^39^ So far, there have been no reports of neutral green electrochromic polymers based on IC units.

With regard to the acceptor in D–A type polymers, electron-withdrawing diketopyrrolopyrrole (DPP) would be a good choice due to the high conjugated lactam structure in the planar backbone which can lead to strong π–π interactions and high charge carrier mobility.^40,41^ Xu group synthesized some of D–A type electrochromic polymers bearing DPP units, which displayed different reversible colors changes even black to transmissive grey, and also presented high optical contrast, short switching time, favorable coloring efficiency and redox stability.^42,43^ Lee *et al.* obtained another DPP-containing conjugated polymers named LGC-D148 which displayed green/transparent electrochromism with the coloration efficiency more than 900 cm^2^ C^−1^.^44^

Inspired by the aforementioned aspects, we synthesized a new kind D–A type electrochromic polymers by Stille polymerization reaction containing 6,12-diphenyl-5,11-dioctyl-5,11-dihydroindolo[3,2-*b*]carbazole as the donor, and 3,6-bis(thienyl-2-yl)-2,5-bis(2-ethylhexyl)-2,5-dihydropyrrolo[3,4-*c*]pyrrole-1,4-dione as the acceptor. The long-chain alkyl linked on IC and DPP units is aim to augment the polymer solubility. In addition, there are 2,2′-bithiophene units in the polymer chain as the bridged groups which could lower the steric hindrance between donor and acceptor, strength π–π interaction, and reduce polymer band gap.^45,46^ By regulating the ratio of donors to acceptors, two polymers namely PDTCZ-1 and PDTCZ-2 respectively were obtained, and the properties of which including electrochemistry, spectroelectrochemistry, dynamics, colorimetry and thermal stability were fully investigated. As expected, the two soluble polymers displayed neutral green electrochromism with small band gaps, and revealed short switching time, desirable coloration efficiency and favorable thermal stability. This is the first report on the indolocarbazole-bearing polymers as neutral green electrochromic material. It is anticipated that the syntheses and characterizations of PDTCZ-1 and PDTCZ-2 will be helpful for providing new materials for the construction of smart windows with commercial values.

## 2. Experimental

### 2.1 Materials

4-Bromobenzaldehyde (99%), indole (98%), tetrabutylammonium bromide (99%), 1-bromooctane (98%), iodine (I_2_, 99.8%), hydrogen iodide (HI, 57%), bis(triphenylphosphine) dichloropalladium (Pd(PPh_3_)_2_Cl_2_, >98%) and tetrabutylammonium hexafluorophosphate (TBAPF_6_, 98%) were purchased from Aladdin Chemical Co., Ltd. (Shanghai, China). Acetonitrile (ACN, 99.9%) was purchased from Tedia Co., Inc. (Fairfield, Ohio, USA). Ethanol, dichloromethane (DCM), hexane, methanol, *N*,*N*-dimethylformamide (DMF), toluene, acetone, chloroform, magnesium sulfate (MgSO_4_, 99.0%), potassium hydroxide (KOH) and sodium hydroxide (NaOH) were purchased from Sinopharm Chemical Reagent Co., Ltd. (Shanghai, China). 5,5′-Bis(trimethylstannyl)-2,2′-bithiophene (>99%), 6-bis(5-bromo-2-thienyl)-2,5-bis(2-ethylhexyl)-2,5-dihydropyrrolo[3,4-*c*]pyrrole-1,4-dione (>98%) were purchased from Derthon Optoelectronic Materials Science Technology Co., Ltd. (Shenzhen, China). The above reagents are used directly without other treatment. indium-tin-oxide (ITO) coated glasses (sheet resistance: <10 Ω sq^−1^) were purchased from Shenzhen CSG Display Technologies.

### 2.2 Polymers syntheses

#### 2.2.1 Syntheses of 6,12-bis(4-bromophenyl)-5,11-dioctyl-5,11-dihydroindolo[3,2-*b*]carbazole

**Scheme 1:**
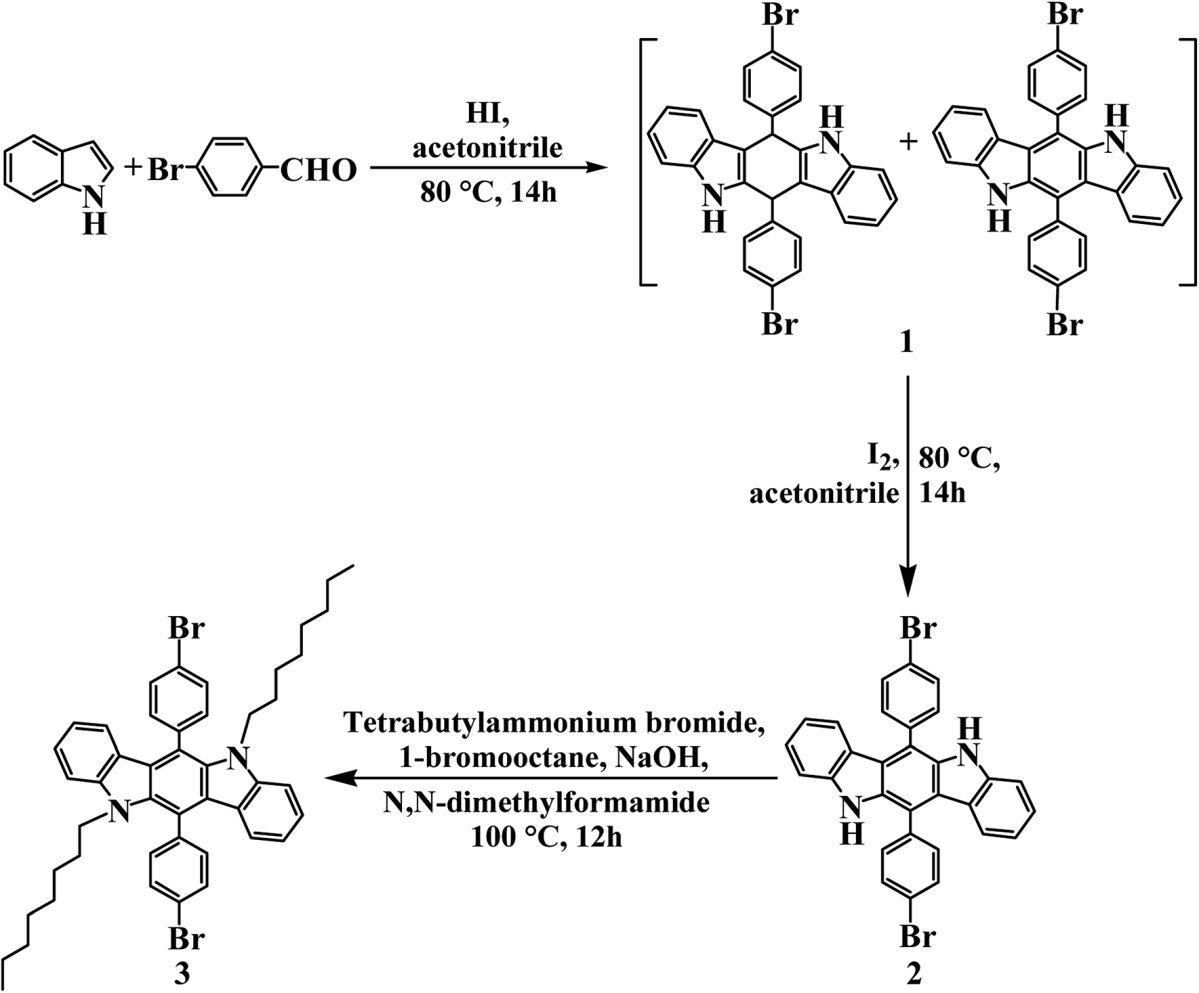
Schematic sketch of synthetic route for 6,12-bis(4-bromophenyl)-5,11-dioctyl-5,11-dihydroindolo[3,2-*b*]carbazole.

The synthetic route was shown in Scheme 1, and the first two steps of reactions were carried out according to ref. 47. In a 250 ml round bottom flask, 2.34 g indole (20 mmol), 3.70 g 4-bromobenzaldehyde (20 mmol) and 1.32 ml HI (57%, 10 mmol) were dissolved in 80 ml ACN. The mixture was refluxed at 80 °C for 14 h. At the end of the reaction, the sediments were filtered out, washed by ACN, and dried under vacuum to obtain product 1. Next, 2.83 g product 1 (5 mmol) and 1.27 g I_2_ (5 mmol) were also dissolved in 80 ml ACN and reacted at 80 °C for 14 h. After vacuum distillation of ACN, 100 ml DCM and 100 ml saturated solution of KOH were put into the remainder, and then the mixture were separated by a pear-shaped funnel to remove I_2_. After drying, filtering and vacuum distilling, the DCM phase was further purified by column chromatography using hexane/DCM (5 : 1, by volume) to gain solid product 2. Finally, 1.70 g product 2 (3 mmol), 2.32 g 1-bromooctane (12 mmol), 0.81 g tetrabutylammonium bromide (2.5 mmol), and 0.74 g NaOH (18.5 mmol) were dissolved in 120 ml DMF, and the mixture were refluxed at 100 °C for 12 hours in nitrogen atmosphere to complete the reaction. Then, the solvent was distilled out, and 100 ml water was added to obtain the precipitate. After filtering and drying, the solid precipitate was purified by column chromatography using hexane/DCM (3 : 1, by volume). The final product 3 6,12-bis(4-bromophenyl)-5,11-dioctyl-5,11-dihydroindolo[3,2-*b*]carbazole was bright yellow powder with 73% yield (1.73 g).^1^H NMR (CDCl_3_, 400 MHz, ppm): *δ* = 7.78 (d, 4H), 7.57 (d, 4H), 7.37 (t, 2H), 7.29 (d, 2H), 6.89 (t, 2H), 6.62 (d, 2H), 3.79 (t, 4H), 1.52 (t, 4H), 1.26 (m, 20H), 0.91 (t, 6H). ^13^C NMR (CDCl_3_, 101 MHz, ppm): *δ* = 142.43, 137.76, 132.31, 132.16, 125.57, 122.19, 118.18, 116.64, 108.39, 77.33, 76.69, 44.55, 31.78, 29.71, 29.27, 29.24, 28.77, 26.81, 22.69, 14.14 (see Fig. S1 in ESI†). Anal. calcd for C_46_H_50_Br_2_N_2_ (%): C, 69.87; H, 6.37; N, 3.54. Found (%): C, 70.93; H, 6.52; N, 3.45.

#### 2.2.2 Syntheses of PDTCZ-1

**Scheme 2:**
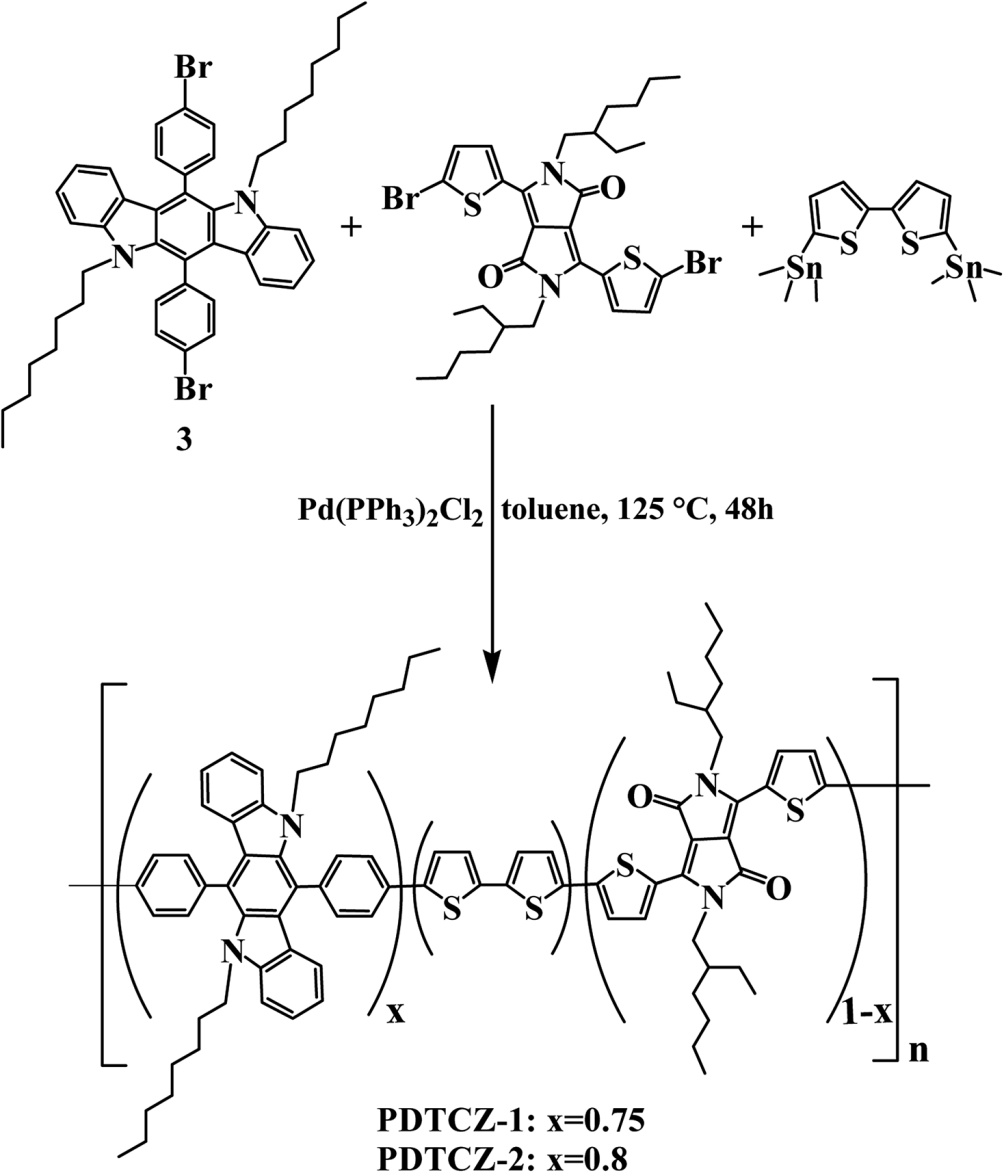
Schematic sketch of synthetic route for PDTCZ-1 and PDTCZ-2.

As shown in Scheme 2, 0.36 g 6,12-bis(4-bromophenyl)-5,11-dioctyl-5,11-dihydroindolo[3,2-*b*]carbazole (0.45 mmol), 0.10 g 6-bis(5-bromo-2-thienyl)-2,5-bis(2-ethylhexyl)-2,5-dihydropyrrolo[3,4-*c*]pyrrole-1,4-dione (0.15 mmol), 0.30 g 5,5′-bis(trimethylstannyl)-2,2′-bithiophene (0.6 mmol), and 0.02 g Pd(PPh_3_)_2_Cl_2_ (0.0285 mmol) were mixed in 120 ml toluene. The above solution was refluxed at 125 °C for 48 hours in argon atmosphere. After distilling toluene by a rotary evaporator and adding 120 ml methanol, the reaction mixture precipitated with sediment. Through filtering and Soxhlet extraction, the green polymer PDTCZ-1 was gained. ^1^H NMR (main signals) (CDCl_3_, 400 MHz, ppm): *δ* = 7.967–6.564 (m, 80H), 4.194–3.673 (m, 16H), 1.628–0.737 (m, 146H), 0.583–0.358 (m, 5H) (see Fig. S2a in ESI†). The GPC data: *M*_w_ = 22.3 kDa, *M*_n_ = 13.4 kDa, polydispersity index = 1.66. Elemental analysis for PDTCZ-1, found (%): C, 71.83; H, 6.15; N, 3.26; S, 10.56.

#### 2.2.3 Syntheses of PDTCZ-2

The preparation method of PDTCZ-2 was the same with that of PDTCZ-1 except for the ratio of reactant. For PDTCZ-2, the detailed reagent amount was 0.38 g 6,12-bis(4-bromophenyl)-5,11-dioctyl-5,11-dihydroindolo[3,2-*b*]carbazole (0.48 mmol), 0.082 g 6-bis(5-bromo-2-thienyl)-2,5-bis(2-ethylhexyl)-2,5-dihydropyrrolo[3,4-*c*]pyrrole-1,4-dione (0.12 mmol), 0.3 g 5,5′-bis(trimethylstannyl)-2,2′-bithiophene (0.6 mmol), and 0.02 g Pd(PPh_3_)_2_Cl_2_ (0.0285 mmol). The product PDTCZ-2 was also green solid. ^1^H NMR (main signals) (CDCl_3_, 400 MHz, ppm): *δ* = 7.998–6.530 (m, 92H), 4.233–3.720 (m, 16H), 1.672–0.695 (m, 176H), 0.503–0.308 (m, 6H) (see Fig. S2b in ESI†). The GPC data: *M*_w_ = 23.8 kDa, *M*_n_ = 13.8 kDa, polydispersity index = 1.72. Elemental analysis for PDTCZ-2, found (%): C, 70.73; H, 6.13; N, 3.19; S, 10.80.

### 2.3 Polymers characterization

^1^H NMR and ^13^C NMR of the products were measured by a Varian AMX 400 MHz spectrometer (Varian Inc., Santa Clara, CA, USA) using CDCl_3_ as solvent and tetramethylsilane as internal standard. Elemental analyses were performed on an elementar vario EL cube elemental analyzer (Elementar Inc., Hanau, Germany). Average molecular weights of the polymers were tested by an alliance GPC 1515 gel permeation chromatography (GPC, Waters Inc., Milford, MA, USA) using tetrahydrofuran as eluent and polystyrene standard as calibrant. Fourier transform infrared (FT-IR) spectra were characterized by a Nicolet 6700 FTIR spectrometer (Thermo Fisher Scientific Inc., Waltham, MA, USA).

To carry out the electrochemical and spectroelectrochemical experiments, the polymer solution was confected using chloroform as solvent (5 mg ml^−1^), and sprayed onto the ITO glasses by an airbrush to form a thin film (the active area: 0.9 cm × 3.0 cm). Electrochemical properties were tested using a CHI 760C electrochemistry workstation (Shanghai Chenhua Instrument Co., Shanghai, China) employing a ITO glasses as working electrode (WE), a platinum ring as counter electrode (CE), and a silver wire (0.03 V *vs.* saturated calomel electrode, SCE) as pseudo-reference electrode (RE) in a self-assembled electrolytic cell.

Spectroelectrochemical experiments were executed by a Varian Cary 5000 spectrophotometer (Varian Inc., Santa Clara, CA, USA). The potentials applied on the polymer was synchronously regulated by the electrochemistry workstation using ITO glass, stainless-steel wire and silver wire as WE, CE and RE, respectively. The Varian Cary 5000 spectrophotometer was also served to record the colorimetric data.

Microscopic morphology was observed by a JSM-6380LV scanning electron microscope (SEM, JEOL Ltd., Tokyo, Japan). Photos of the polymer solution and film were obtained by a Canon digital camera (Canon Inc., Tokyo, Japan). Thermogravimetric analysis (TGA) was surveyed by a Netzsch STA449C TG/DSC simultaneous thermal analyzer (Netzsch Inc., Bavaria, Germany) with 10 °C min^−1^ heating rate from 0 to 750 °C in nitrogen atmosphere.

## 3. Results and discussion

### 3.1 FT-IR spectra

**Fig. 1:**
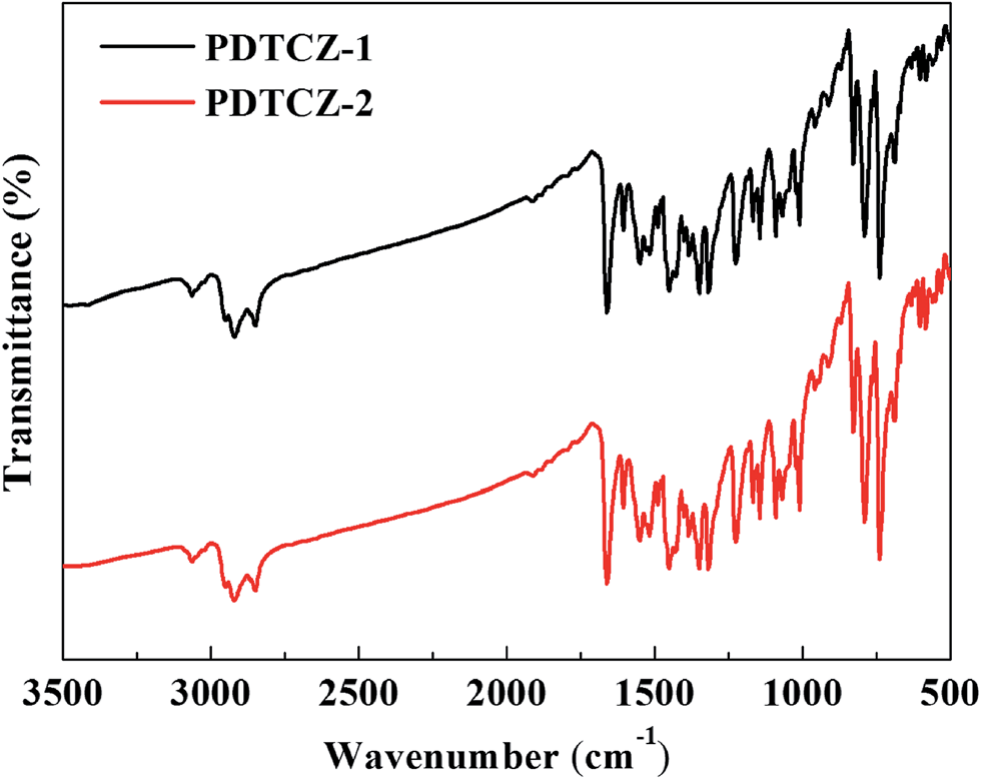
FT-IR spectra of PDTCZ-1 and PDTCZ-2.

FT-IR spectra can be used to analyze the type of functional groups and deduce the structure of compound, which were measured for the two polymers and shown in Fig. 1. The following peaks were identified in the spectrum of PDTCZ-1: 3063 cm^−1^ (aromatic C–H stretching vibration), 2922 cm^−1^ and 2851 cm^−1^ (aliphatic C–H stretching vibration), 1661 cm^−1^ (ketone C=O stretching vibration), 1603 cm^−1^, 1550 cm^−1^, 1515 cm^−1^ and 1488 cm^−1^ (benzene, thiophene, IC and DPP substituted ring C=C stretching vibration), 1450 cm^−1^, 1430 cm^−1^, 1400 cm^−1^ and 1382 cm^−1^ (methylene and methyl C–H bending vibration), 1352 cm^−1^, 1318 cm^−1^, 1225 cm^−1^, 1169 cm^−1^ and 1145 cm^−1^ (benzene, thiophene, IC and DPP substituted ring C–C, C–S and C–N stretching vibration), 1090 cm^−1^, 1070 cm^−1^ and 1010 cm^−1^ (benzene, thiophene and IC substituted ring C–H in-plane bending vibrations), 828 cm^−1^, 790 cm^−1^, 739 cm^−1^ (benzene, thiophene and IC substituted ring C–H out of plane bending vibrations), 690 cm^−1^ (methylene C–H out of plane bending vibrations). It was observed that PDTCZ-2 presented almost the same characteristic absorption peaks compared with PDTCZ-1 except for the slight difference in the peak intensity, which could be explicated by the different proportion of donors to acceptors in the polymer backbone.

### 3.2 Electrochemistry

**Fig. 2:**
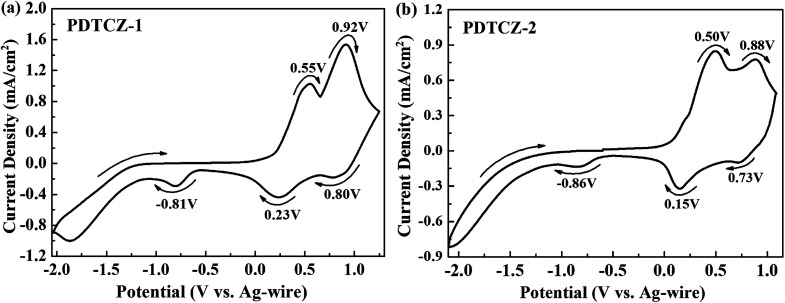
Cyclic voltammetry (CV) curves of (a) PDTCZ-1 and (b) PDTCZ-2.

Electrochemical properties of the two polymer films were determined by cyclic voltammetry (CV) experiments which were executed in ACN solution (including 0.1 M TBAPF_6_ as electrolyte) at 100 mV s^−1^ scan rate. From CV curves shown in Fig. 2, it can be seen that PDTCZ-1 and PDTCZ-2 displayed the similar redox procedures. Specifically, they displayed two pairs of oxidation–reduction peaks during the p-type doping which were situated respectively at 0.55 V and 0.23 V as well as 0.92 V and 0.80 V for PDTCZ-1, and at 0.50 V and 0.15 V as well as 0.88 V and 0.73 V for PDTCZ-2. And the two oxidation peaks was resulted from the formation of polarons and bipolarons.^21,44^ The onset oxidation potentials (*E*_onset_) of PDTCZ-1 and PDTCZ-2 were evaluated as 0.19 V and 0.15 V, respectively. Based on the above data, it was easy to recognize that the oxidation–reduction peak potentials and *E*_onset_ of PDTCZ-1 were higher than those of PDTCZ-2 as a result of the different ratio of donors to acceptors in the polymer main chain. In the D–A type structure, the increasing of donor units would be of benefit to raise the HOMO energy level of the polymer, and in turn reduce the potential required for the formation of radical cation.^48^ Consequently, PDTCZ-2 containing more donor units showed lower redox potentials and *E*_onset_ than PDTCZ-1. Besides, each polymer exhibited individually a slight reduction peak at −0.81 V for PDTCZ-1 and −0.86 V for PDTCZ-2 under the negative potentials, representing the inconspicuous n-type doping process, which might because the presence of little water and dissolved oxygen in the electrolyte solution made it difficult for an inherently unstable n-type doping to exist.^49^

### 3.3 Morphology

**Fig. 3:**
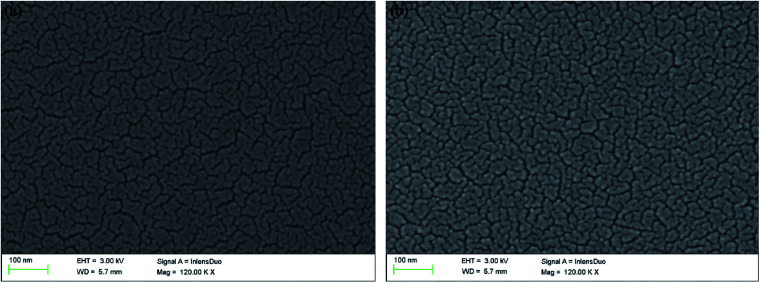
Scanning electron microscopy (SEM) images of (a) PDTCZ-1 and (b) PDTCZ-2.

Using JSM-6380LV scanning electron microscope, the morphology was clearly presented with 120 000 times magnification after the polymers were spray-coated onto the ITO glasses. As shown in Fig. 3(a), on the surface of PDTCZ-1, many curving texture structures like the capillaries of human body were arranged uniformly, among which a large number nano-scale particles of the polymer were distributed with irregular shape. This microstructure was due to the cracking of thin film resulted from the volatilization of solvent during the preparation of polymer membrane. The presence of these nano-sized cracks would be conducive to the doping and de-doping of ions, thus increasing the optical contrast and accelerating the speed of electrochromic switch.^43^ The morphology of PDTCZ-2 (Fig. 3(b)) was very similar to that of PDTCZ-1, except that more polymer particles were dispersed throughout the surface and surrounded by more concentrated stripes. This phenomenon could also be interpreted by the different donor/acceptor ratio which might lead to different degree of cracking in the volatilization process.

### 3.4 Optical properties

**Fig. 4:**
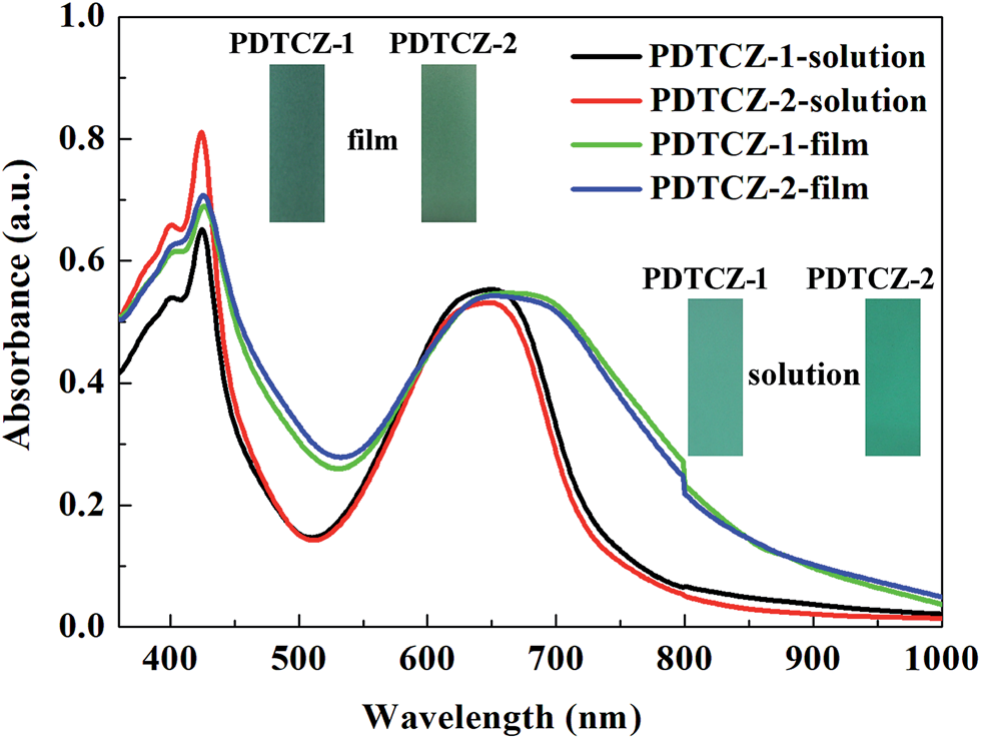
UV-vis absorbance spectra of the polymers in solution and in thin film.

PDTCZ-1 and PDTCZ-2 were dissolved in chloroform respectively to form their saturated solution, and part of solution was sprayed onto the ITO glasses to prepare thin films. The optical properties of the polymers in both liquid and solid phases were detected and shown in Fig. 4. Both in chloroform solution and in solid film, the two polymers exhibited two distinct absorption bands centered around 425 nm and 650 nm which were ascribed to the high energy and the low energy π–π* transitions.^50^ And the absorption peaks was considered to be caused by the transitions from IC and thiophene based valence bands to their antibonding counterparts (high-energy transition) and to the substituent localized narrow conduction bands (low-energy transition).^51^ It was also seen clearly that the positions of absorption peaks of PDTCZ-1 and PDTCZ-2 were almost identical in the same phase state which were located at 424 nm and 645 nm in the solution, as well as 426 nm and 660 nm in the film, respectively. What was noteworthy was that the absorption peaks of the two polymers showed obvious bathochromic shifts and broadening phenomena in the solid state in contrast with those in the solution state especially for the low energy peaks, which demonstrated the enhanced π–π* stacking and stronger intra molecular interaction in the solid polymer films.^43,52^ Also because the centers of absorption bands were all located at about 425 nm and 650 nm, the colors of the two polymers were displayed as green. In the solution, PDTCZ-1 and PDTCZ-2 showed light emerald and deep emerald respectively, while in the solid film, PDTCZ-1 and PDTCZ-2 presented sea-green and forest-green respectively.

### 3.5 Spectroelectrochemistry

**Fig. 5:**
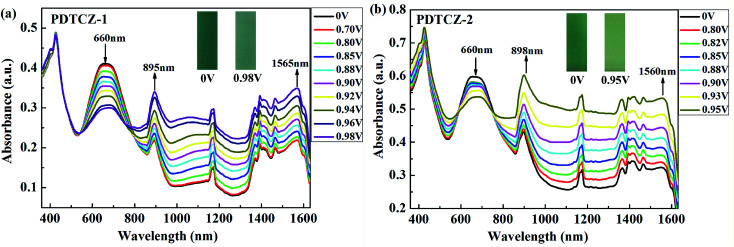
Spectroelectrochemical spectra of (a) PDTCZ-1 film and (b) PDTCZ-2 film.

The two polymers were spray-coated onto the ITO glasses and performed spectroelectrochemical tests to investigate the evolution of charge carries during the oxidation course. The experiments were conducted in the 0.1 M TBAPF_6_/ACN solution at the potential from 0 V to 0.98 V for PDTCZ-1 and from 0 V to 0.95 V for PDTCZ-2 with the irregular intervals. The detailed absorption spectra as well as the colors of polymer films in the neutral and oxidized states were shown in Fig. 5.

Both PDTCZ-1 and PDTCZ-2 manifested two absorption peaks in the neutral state centered at 426 nm and 660 nm corresponding to the π–π* transitions in the polymer backbone. When the applied potentials increased gradually, the absorption bands at 660 nm weakened gradually, but those at 426 nm remained largely unchanged. Concurrently, other two new absorption bands emerged and heightened in the near infrared region (NIR) owing to the formation of polarons and bipolarons.^21,38^ It could be observed from Fig. 5(a) that the absorption band around 660 nm of PDTCZ-1 attenuated gradually when fully oxidized to 0.98 V, meanwhile, the polaron and bipolaron bands augmented constantly centered at 895 nm and 1565 nm, respectively. With regard to PDTCZ-2 as shown in Fig. 5(b), it exhibited the similar changing trend to PDTCZ-1, except that the polaron and bipolaron bands were located at 898 nm and 1560 nm respectively. Due to the fading of the high-energy absorption peaks in the visible region (did not vanish completely) and the intensifying of the low-energy absorption peaks in the NIR, the polymers demonstrated corresponding color changes from sea-green to pale green for PDTCZ-1 and from forest-green to pale yellowish-green for PDTCZ-2.

**Fig. 6:**
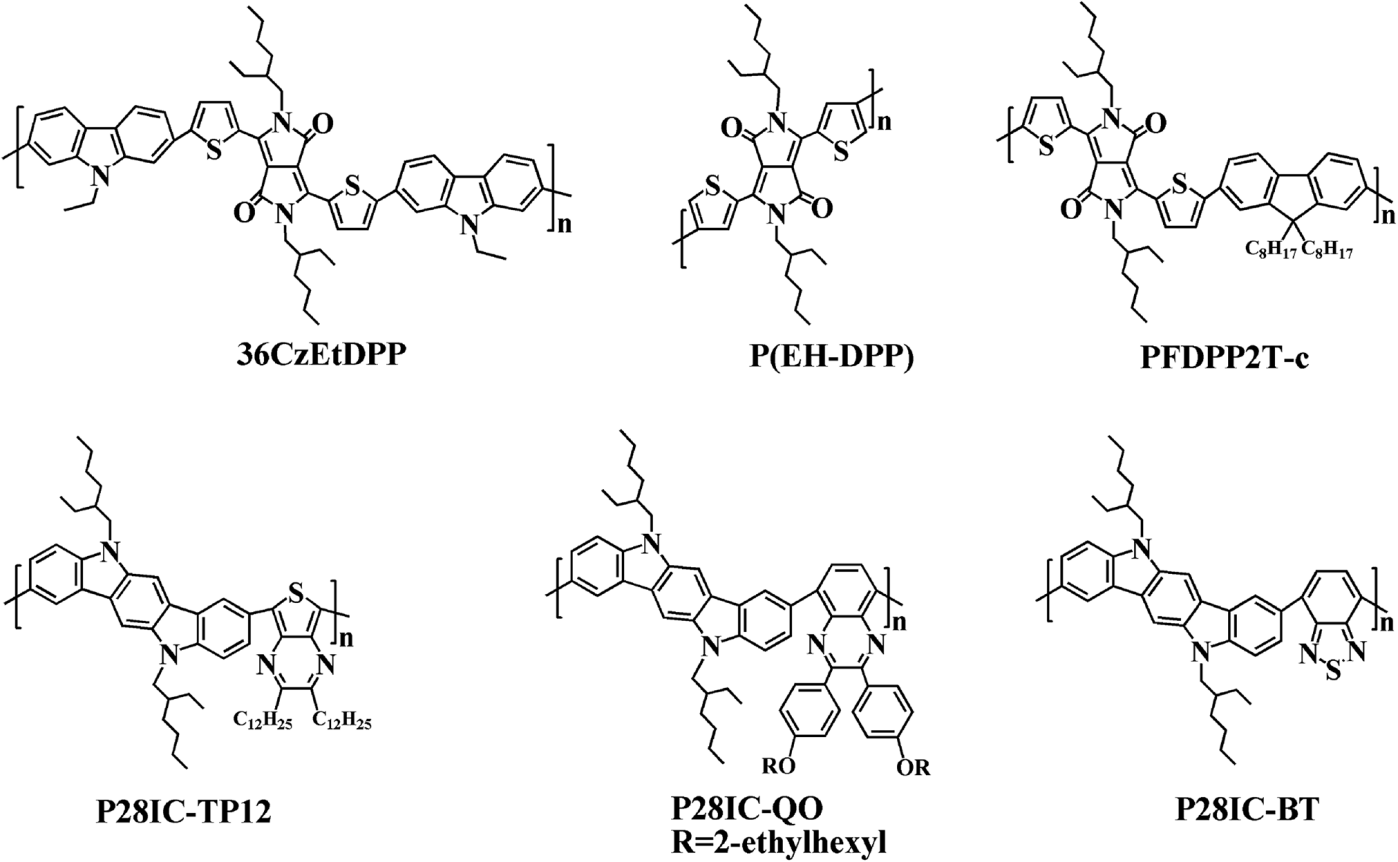
Molecular structures of some donor–acceptor (D–A) type polymers containing DPP or IC units.

In addition, the low energy onset absorption wavelengths (*λ*_onset_) of the polymer films in the neutral state were measured as 861 nm for PDTCZ-1 and 893 nm for PDTCZ-2. Taking advantage of the values of *λ*_onset_ and *E*_onset_, the optical band gaps (*E*_g,op_), as well as the HOMO and LUMO energy levels were calculated, and these values mentioned above together with the maximum absorption wavelengths (*λ*_max_) were all listed in Table 1. The *E*_g,op_ of PDTCZ-1 and PDTCZ-2 were 1.44 eV and 1.39 eV respectively, which were lower than that of other DPP or IC units containing D–A polymers. The spectroscopic parameters and molecular structures of the compared polymers were illustrated in Table 1 and Fig. 6. As demonstrated in the table, three kinds of polymers bearing DPP units as acceptor, including P(36CzEtDPP) with carbazole units as donor,^40^ P(EH-DPP) with thiophene units as donor,^53^ and PFDPP2T-c with fluorene units as donor,^54^ revealed the *E*_g,op_ in the range of 1.72–1.76 eV. While other three kinds of polymers containing IC units as donor, such as P28IC-TP12 with thieno[3,4-*b*]pyrazine units as acceptor, P28IC-QO with quinoxaline units as acceptor and P28IC-BT with benzothiadiazole units as acceptor, exhibited the higher *E*_g,op_ between 1.89–2.34 eV.^55^ By comparison, PDTCZ-1 and PDTCZ-2 displayed the lowest *E*_g,op_ (1.44 eV and 1.39 eV), which confirmed the high matching between DPP unit and IC unit along with the effective hybridization of the HOMO and LUMO energy level.

**Table 1:** Electrochemical and optical parameters of PDTCZ-1 and PDTCZ-2

Polymer	*λ*_max_ (solution) (nm)	*λ*_max_ (film) (nm)	*λ*_onset_ (film) (nm)	*E*_onset_ (V)	*E*_g,op_ a (eV)	HOMO^b^ (eV)	LUMO^c^ (eV)
PDTCZ-1	424/645	426/660	861	0.19	1.44	−4.90	−3.46
PDTCZ-2	424/645	426/660	893	0.15	1.39	−4.86	−3.47
P(36CzEtDPP)^d^	—	596	720	0.43	1.72	−5.23	−3.74
P(EH-DPP)^e^	—	—	—	0.81	1.76	−5.81	−4.05
PFDPP2T-c^f^	658	656	701	—	1.77	—	—
P28IC-TP12^g^	—	347/557	—	0.56	1.89	−4.88	−2.99
P28IC-QO^g^	—	367/445	—	0.76	2.34	−5.09	−2.75
P28IC-BT^g^	—	332/430/516	—	0.58	2.09	−4.90	−2.81

a *E*_g,op_ = 1241/*λ*_onset_.

b HOMO = −*e*(*E*_onset_ + 4.71).

c LUMO = HOMO + *E*_g,op_.

d Data from ref. 40.

e Data from ref. 53.

f Data from ref. 54.

g Data from ref. 55.

Moreover, compared with PDTCZ-1, PDTCZ-2 displayed lower *E*_g,op_ resulting from the larger proportion of donor units in its backbone. It could be found that the calculated HOMO energy level of PDTCZ-2 was higher than that of PDTCZ-1, which was consistent with the previous conclusion that more donor units in the polymer was benefit to improve the HOMO energy level. However, the LUMO energy levels of PDTCZ-1 and PDTCZ-2 were hardly changed. For reasons given above, PDTCZ-2 showed the lower *E*_g,op_ than PDTCZ-1.

As mentioned earlier, neutral green polymer, serving as the RGB (red, green, blue) electrochromic material, is relatively difficult to achieve. In particular, the green-color electrochromic polymers with good solubility are reported seldom in the literatures. Such soluble neutral green polymers usually included different donor/acceptor combination such as thiophene/thienopyrazine,^23^ ProDOT/benzothiadiazole,^25^ EDOT/quinoxaline,^26^ ProDOT/benzoselenadiazole,^20^ and cyclopentadithiophene/benzothiadiazole.^17^ However, IC units had never been employed as donor to prepare soluble neutral green electrochromic polymers in the previous reports. In this study, two IC-containing polymers with neutral green color, good solubility, and low band gap were prepared for the first time, which had important theoretical significance for the development of neutral green electrochromic polymers.

### 3.6 Electrochromic switching

**Fig. 7:**
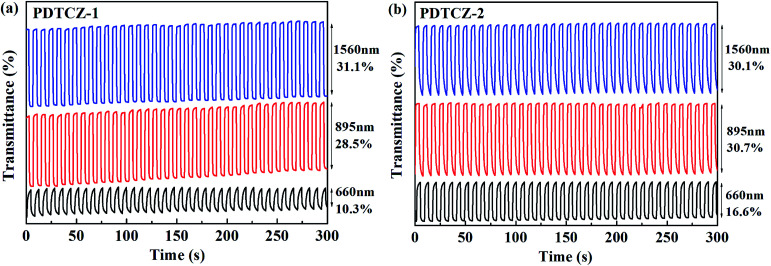
Electrochromic switching of (a) PDTCZ-1 and (b) PDTCZ-2.

The kinetic parameters, as the very important performance characterization for the electrochromic materials, could be obtained employing the spectrophotometer combined with the electrochemical workstation by double potential step chronoamperometry. After the polymers were spray-coated onto the ITO glasses, the experiments were carried out under the square wave potential with the orderly 4 s interval between 0 V and 1.1 V in 0.1 M TBAPF_6_/ACN solutions. The measurement wavelengths were chosen around *λ*_max_ of the polymer films in the visible region and the NIR according to the spectroelectrochemical experiments, located at 660 nm, 895 nm and 1560 nm for PDTCZ-1 and PDTCZ-2. The spectra of transmittance changes from 0 to 300 s at different wavelengths and three kinds of dynamic parameters were illustrated in Fig. 7 and Table 2, respectively.

**Table 2:** Optical contrast (*T*%), response time (*t*_95%_) and coloration efficiency (CE) of PDTCZ-1 and PDTCZ-2

Polymer	*λ* (nm)	Δ*T*% (%)	*t*_95%_ (s)	CE (cm^2^ C^−1^)
PDTCZ-1	660	10.3	2.84	89.0
	895	28.5	0.53	141.0
	1560	31.1	0.73	197.8
PDTCZ-2	660	16.6	2.50	96.6
	895	30.7	2.18	168.7
	1560	30.1	2.73	91.6

Optical contrast (*T*%), means percentage change in transmittance between the redox state, was measured as 10.3% at 660 nm, 28.5% at 895 nm and 31.1% at 1560 nm for PDTCZ-1, and as 16.6% at 660 nm, 30.7% at 895 nm and 30.1% at 1560 nm for PDTCZ-2. The two polymers presented higher *T*% in the NIR than in the visible region, which was coincident with many previous reported D–A type polymers.^29,30^ On the other hand, by comparing with each other, PDTCZ-2 indicated high *T*% than PDTCZ-1 especially in the visible region because the larger amounts of IC units as donors in PDTCZ-2 backbone might enhance the susceptibility toward electrochemical oxidation, which correlated well with its higher HOMO energy level as mentioned above.

Response time (*t*_95%_) refers to the time necessary for 95% of full optical switching during the redox process, which could represent the electrochromic velocity. The calculated values of *t*_95%_ were 2.84 s at 660 nm, 0.53 s at 895 nm and 0.73 s at 1560 nm for PDTCZ-1, and 2.50 s at 660 nm, 2.18 s at 895 nm and 2.73 s at 1560 nm for PDTCZ-2. The *t*_95%_ at all test wavelengths were below 3 s, suggesting that the two polymers possessed relatively fast switching speed, particularly for PDTCZ-1 which showed 0.53 s and 0.73 s respectively in the NIR.

Coloration efficiency (CE), using to estimate the utilization efficiency of energy in the electrochromic switching process, can be expressed as the ratio between optical density change (ΔOD) and charge consumed per unit electrode area (Δ*Q*) and obtained by the following formulas:^44^
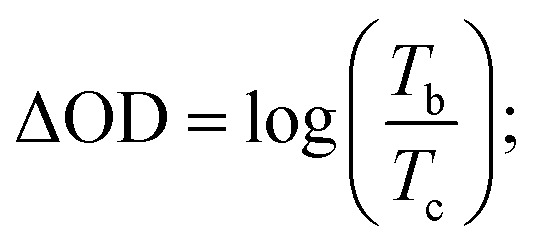

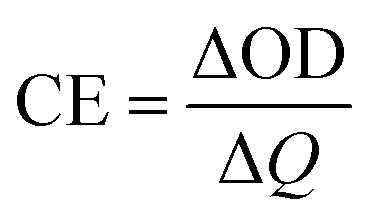
where *T*_b_ and *T*_c_ mean the transmittance before and after coloration, respectively. The values of CE were calculated as 89.0 cm^2^ C^−1^ at 660 nm, 141.0 cm^2^ C^−1^ at 895 nm and 197.8 cm^2^ C^−1^ at 1560 nm for PDTCZ-1, and as 96.6 cm^2^ C^−1^ at 660 nm, 168.7 cm^2^ C^−1^ at 895 nm and 91.6 cm^2^ C^−1^ at 1560 nm for PDTCZ-2. The higher CE values occurred in the NIR compared with those in the visible region for the two polymers except for a lower CE of PDTCZ-2 at 1560 nm. In summary, the opinion of optimum donor-to-acceptor proportion for conjugated polymer should be supported, which deserved further investigations to reveal more regularity in the electrochromic properties.

**Fig. 8:**
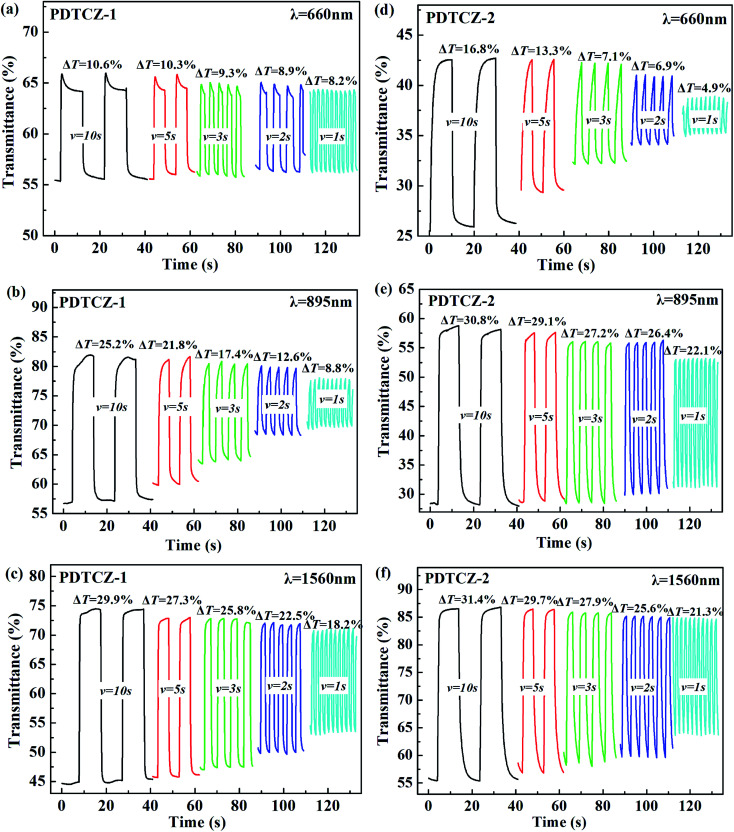
Electrochromic switching of (a) PDTCZ-1 at 660 nm, (b) PDTCZ-1 at 895 nm, (c) PDTCZ-1 at 1560 nm, (d) PDTCZ-2 at 660 nm, (e) PDTCZ-2 at 895 nm, and (f) PDTCZ-2 at 1560 nm with different time intervals.

In order to test the cyclic stability of the polymers in electrochromic process, the time intervals of square wave potentials in dynamic experiments were set to 10 s, 5 s, 3 s, 2 s, and 1 s respectively to provide the relations of optical contrast *versus* time. As demonstrated in Fig. 8, when the retention time changed from 10 s to 1 s, the *T*% of PDTCZ-1 decreased by 2.4% at 660 nm, 16.4% at 895 nm and 11.7% at 1560 nm, and that of PDTCZ-2 decreased by 11.9% at 660 nm, 8.7% at 895 nm, and 10.1% at 1560 nm. It was observed that the *T*% was greatly dependent on the switching time and the two polymers showed various degree of decrease in *T*% at different wavelengths.^42,56^ With the decease of the retention times, the polymers could not be fully doped (oxidized) or dedoped (reduced), resulting in the decrease in the contrast ratios (*T*%).^57^ On another aspect, these data suggested that PDTCZ-1 and PDTCZ-2 were hardly the best materials to be display-type devices, but might be the potential candidates in the applications of smart windows and other devices.

### 3.7 Colorimetry

To further distinguish the similar color changes of green for PDTCZ-1 and PDTCZ-2, the values of L*, a*, and b* were determined by colorimetric experiments which were conducted in the 0.1 M TBAPF_6_/ACN solution at the scanning potential from 0 to 1.3 V using CIE 1976 L* a* b* Color Space software after the polymers were spray-coated onto the ITO glasses. L* symbolizes the luminance of color from black to white, a* symbolizes the contrast of red *versus* green, and b* symbolizes the contrast of yellow *versus* blue.^25,56^

**Fig. 9:**
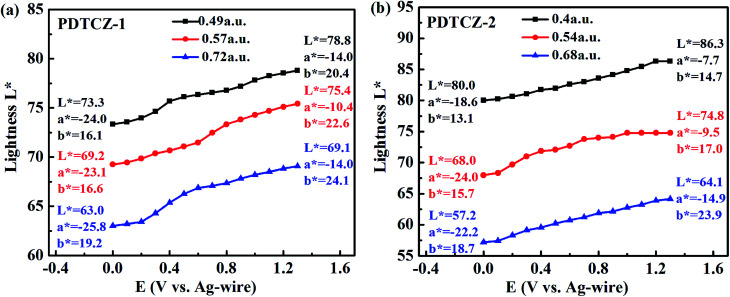
Plot of lightness of (a) PDTCZ-1 and (b) PDTCZ-2 as a function of applied potential.

Three thin films of each polymer with different thickness participated in the experiments, and the thickness was described by the maximum optical absorption value, that is, the larger the value was, the thicker the film was. Fig. 9 depicted the variations of L* with increasing potentials and listed the values of L*, a*, and b* at 0 V and 1.3 V respectively. As shown in the figure, it was not until the applied potentials exceeded *E*_onset_ that the L* values of the two polymers began to change obviously. And as the applied potentials continued to raise, the L* of PDTCZ-1 and PDTCZ-2 gradually enhanced to the maximum at 1.3 V. Besides, the values of a* improved from the larger negatives to the smaller negatives when the polymer films changed from the neutral state to the oxidation state, meanwhile, the values of b* increased for certain degree in the positive range, which suggested the fading of green and the dyeing of yellow in the oxidation process just like the images of the two polymers showed in Fig. 5.

With the thickness of the same polymer (*i.e.* the maximum optical absorption value) increasing, the L* values decreased during the whole applied potentials. For instance, the L* values of PDTCZ-1 were measured as 73.3, 69.2 and 63.0 at 0 V, and as 78.8, 75.4 and 69.1 at 1.3 V when the maximum absorption grew from 0.49 to 0.57, and to 0.72 a.u. PDTCZ-2 revealed the similar decrease of the L* values as PDTCZ-1. As the thickness of the polymer films increased, the intensity of the light passing through the films decreased, resulting in the weakening of the luminance and the decrease of the L* value. Therefore, the luminance could be tuned by controlling the polymer film thickness, which had great practical significance for the application of electrochromic devices.

### 3.8 Thermogravimetric analysis

**Fig. 10:**
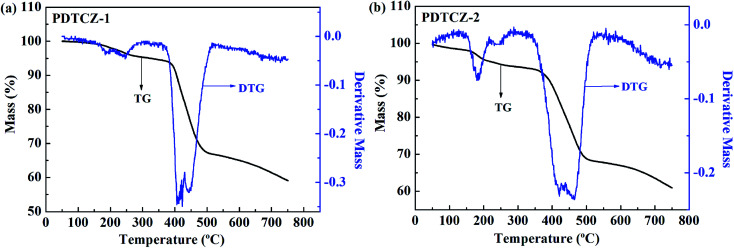
Thermogravimetric analysis (TGA) of (a) PDTCZ-1 and (b) PDTCZ-2.

For an excellent electrochromic material, it is very pivotal to have good thermal stability which can be characterized by TGA test.^55^ As shown in Fig. 10, it was found from the thermogravimetric (TG) curves that there was a small amount of weight loss before about 150 °C for the two polymers, which might be due to the evaporation of residual solvent or water in the polymers. Then, the polymers continued to suffer small weight loss before about 380 °C owing to the decomposition of partial alkyl chains in the polymer backbones, and this process was also reflected by the weaker peaks of the differential thermal gravity (DTG) curves. The decomposition temperature at 5% weight loss (*T*_d_) of PDTCZ-1 and PDTCZ-2 were measured as 324.6 °C and 221.5 °C, respectively. Significant decomposition occurred approximately between 389.3 °C (extrapolated onset temperature, *T*_onset_) and 482.7 °C (extrapolated termination temperature, *T*_offset_) for PDTCZ-1, as well as between 383.3 °C (*T*_onset_) and 492.5 °C (*T*_offset_) for PDTCZ-2. It was observed from the DTG curves that the temperatures with maximum degradation rate (*T*_max_) were measured as 422.9 °C for PDTCZ-1 and as 421.7 °C for PDTCZ-2. The amount of carbonized residue, also known as char yield (% char), were 59.1% for PDTCZ-1 and 60.9% for PDTCZ-2 at 750 °C, which was attributed to a large number of aromatic groups distributed in the polymer backbone. The thermogravimetric parameters of *T*_d_, *T*_onset_, *T*_offset_, *T*_max_ and % char were all listed in Table 3. According to the above data, both PDTCZ-1 and PDTCZ-2 exhibited desirable thermal stability, which would in turn be propitious to improve the morphological stability and the service time in the practical application.

**Table 3:** The thermogravimetric parameters of PDTCZ-1 and PDTCZ-2

Polymer	*T*_d_ (°C)	*T*_onset_ (°C)	*T*_offset_ (°C)	*T*_max_ (°C)	% char
PDTCZ-1	324.6	389.3	482.7	422.9	59.1
PDTCZ-2	221.5	383.3	492.5	421.7	60.9

## 4. Conclusions

In this work, two soluble IC-containing polymers with neutral green electrochromism, *i.e.* PDTCZ-1 and PDTCZ-2, were reported for the first time. As the D–A type polymers, they were obtained by Stille crossing-coupling reactions using IC units as donor and DPP units as acceptor. Both PDTCZ-1 and PDTCZ-2 were the neutral green polymers with low optical band gaps and favorable thermal stability, and also displayed relatively high optical contrast and satisfactory coloration efficiency especially in the NIR. Moreover, the feed ratio of donors to acceptors proved to be an impactful technique to regulate the electrochromic properties which were manifested in the lower onset oxidation potential and optical band gap of PDTCZ-2 containing larger proportion of donor units in comparison with PDTCZ-1. In conclusion, PDTCZ-1 and PDTCZ-2 displayed the individually distinguishing feature, and more important, they provided a new structure design for green electrochromic materials employing IC units as donor, which made it necessary to research other IC-containing polymers and develop their applications further.

## Conflicts of interest

There are no conflicts to declare.

## Supplementary Material

RA-008-C8RA03552K-s001
