# Supplementary material for: Synthesis and characterization of novel donor–acceptor type neutral green electrochromic polymers containing an indolo[3,2-b]carbazole donor and diketopyrrolopyrrole acceptor
Source: RSC Adv. 2018 Jun 11;8(38):21252–64. doi: 10.1039/c8ra03552k (PMC9080953; doi:10.1039/c8ra03552k)
Supplement: RA-008-C8RA03552K-s001 [file RA-008-C8RA03552K-s001.pdf]

Electronic Supplementary Material (ESI) for RSC Advances.
This journal is © The Royal Society of Chemistry 2018

Electronic supplementary information

Yan Zhang, [a] Lingqian Kong, [b] Xiuping Ju[b] Hongmei Du[a], Jinsheng Zhao*[a], Yu Xie*[c]

[a]Shandong Key Laboratory of Chemical Energy Storage and Novel Cell Technology, Liaocheng University, Liaocheng, 252059, P. R. China.

[b]Dongchang College, Liaocheng University, Liaocheng, 252059, P. R. China.

[c]College of Environment and Chemical Engineering, Nanchang Hangkong University, Nanchang 330063, PR China.

*Correspondence: j.s.zhao@163.com (Jinsheng Zhao); xieyu_121@163.com (Yu Xie)

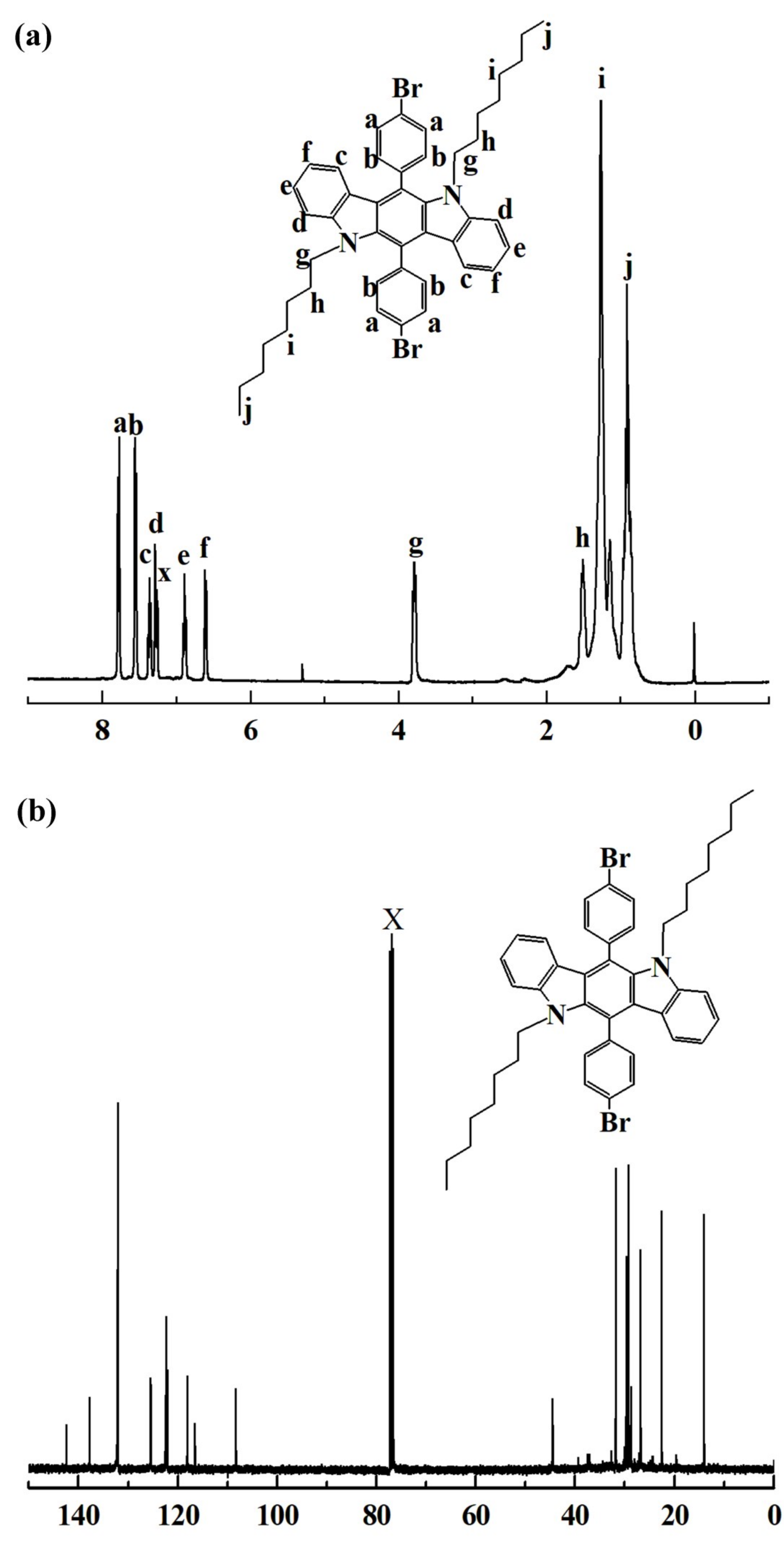

(a)
Br
N
N
Br
a b c d e f g h i j
ab
c
d
x
e
f
g
h
i
j
8
6
4
2
0
(b)
X
Br
N
N
Br
140
120
100
80
60
40
20
0

Figure S1. (a) ^{1}H NMR spectrum of 6,12-bis(4- bromophenyl)-5,11-dioctyl-5,11-dihydroindolo[3,2-b]carbazole in $CDCl_3$, solvent peak at δ = 7.26 ppm was marked by "x", (b) ^{13}C NMR spectrum of 6,12-bis(4- bromophenyl)-5,11-dioctyl-5,11-dihydroindolo[3,2-b]carbazole in $CDCl_3$, solvent peak at δ = 77.01 ppm was marked by "X".

(a) PDTCZ-1

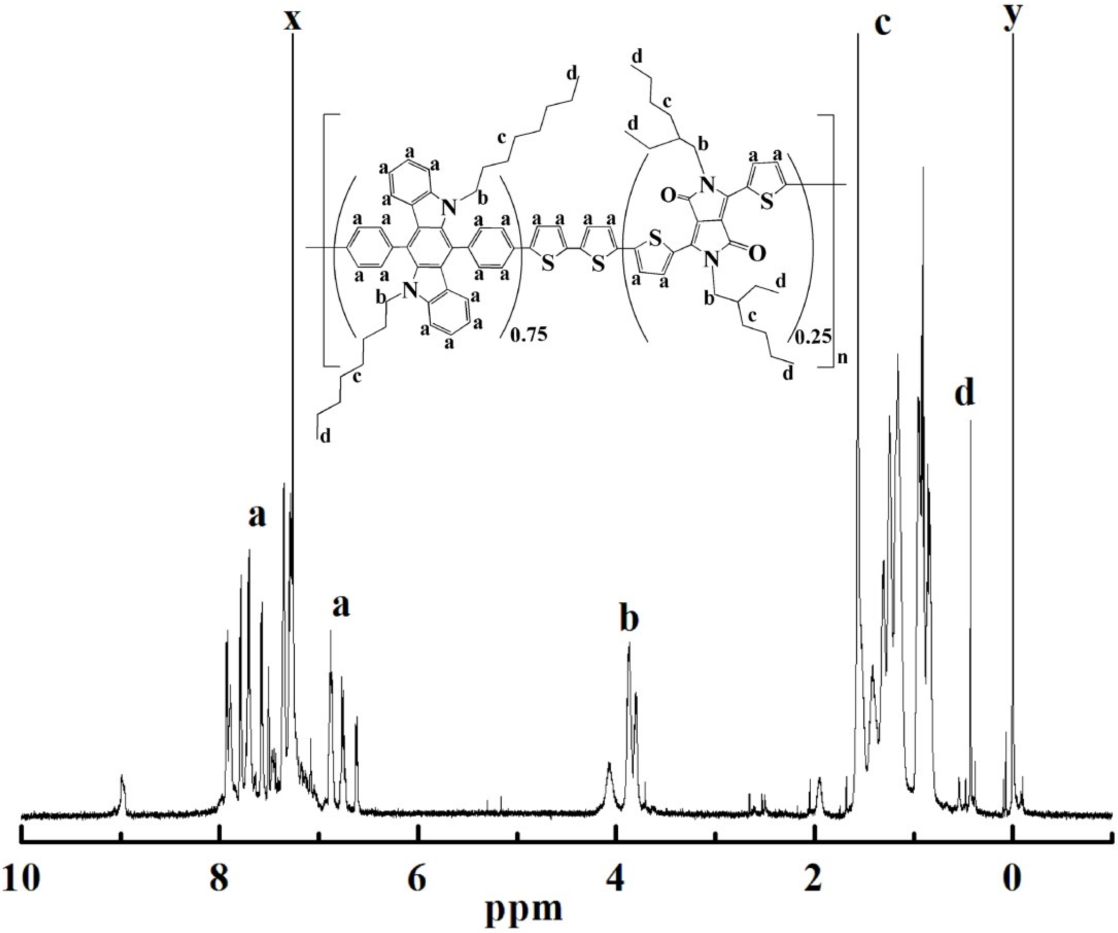

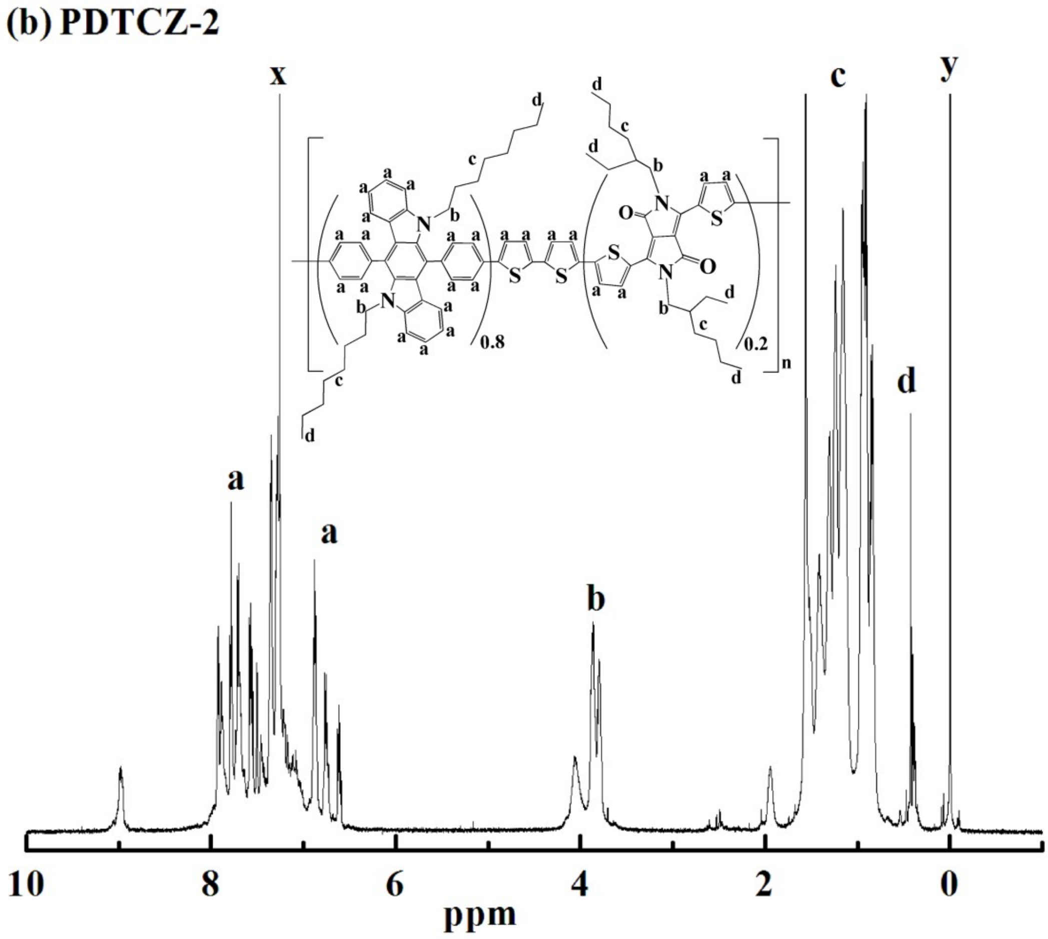

Figure S2. ^{1}H NMR spectra of (a) PDTCZ-1 and (b) PDTCZ-2 in $CDCl_3$. Solvent and tetramethylsilane peaks were marked by “x”, “y” respectively.
